# Book Reviews and Notices

**Published:** 1888-06

**Authors:** 


### BOOK REVIEWS AND BOOK NOTICES.

---

The Throat and Its Diseases, Including Associated Affections of the Nose and Ear.

With 120 illustrations in color and 200 engravings, designed and executed by the author, LENNOX BROWNE, F. R. C. S. E., Senior Surgeon to the Central London Throat and Ear Hospital, Surgeon and Aural Surgeon to the Royal Society of Musicians, Consulting Surgeon to the Newcastle Throat and Ear Hospital, etc. Second edition, rewritten and enlarged. Pp. xviii, 614. Philadelphia: Lea Brothers & Co. Chicago: A. C. McClurg & Company. 1887.

The first edition of Mr. Lennox Browne's work on "The Throat and its Diseases," which appeared in 1879, was limited in design to the consideration of diagnosis and treatment. This strictly clinical aspect of the case was, however, presented in a style so precise and forcible that a desire was at once excited for an equally lucid and authoritative treatment of associated pathological states and questions. The book is now before us in its second edition, rewritten, enlarged, and with the pathological deficiency abundantly provided for. The author has for twenty years enjoyed exceptional clinical advantages, and as a laryngologist ranks a peer among his fellows of the English metropolis. Truly literate, possessed of broad ideas, a sound judgment, a high regard for the practical, and withal an artist of considerable ability, he has succeeded in infusing an individuality into his book which makes the work truly his own.

The first eight chapters are devoted to the special anatomy and physiology of the larynx, pharynx, and nose, and to general diagnosis, semiology, and therapeutics, including consideration of electrical and lime-light illumination, photo-laryngoscopy, etc. Dissatisfaction is expressed with all forms of electrical illuminating apparatus yet devised, preference being accorded to the oxyhydrogen lime-light when extra illumination is necessary. Prominence is given to Leiter's leaden coil as a temperature regulator. It is readily adapted to the form of the neck, and serves as an elegant substitute for the poultice, and especially the troublesome ice-bag, as a means of subduing acute inflammation of the tonsils, pharynx, or larynx.

It is to be regretted that in the consideration of chronic pharyngitis, the various forms of this disease had not been more carefully differentiated. Doubtless the hypertrophic, the atrophic, and the simple chronic pharyngitis are pathologically stages of the same imflammation, yet clinically, and especially from a therapeutic point of view, they are so distinct that word-pictures of each were desirable.

Varix of the base of the tongue, associated with lymphoid hypertrophies at the same point, the author thinks accountable for many pharyngeal paræsthesiæ which are often erroneously attributed to hysteria.

So much nonsense has been informally promulgated concerning the operation of tonsilotomy that we quote the following passage from this authority: "Chronic enlargment of the tonsils is only to be treated satisfactorily by the one method of excision, and there does not appear any valid reason why there should be two opinions on the subject." It is assumed, of course, that the operation be skillfully performed. The chapters on "Cancer of the Tonsil" and on "Lupus of the Mouth, Pharynx, and Larynx," are unique and deserving of careful study.

Mr. Browne enters a powerful plea in favor of the non-identity of croup and diphtheria. Attention is drawn to the fact that false membranes of the same macroscopic and microscopic character as those of diphtheritic origin can be produced on the mucous lining of the air-passages by every kind of traumatism. A lady, by accident, received some eau de Cologne into her trachea by her nostril. Membrane was profusely developed on the third day, and on the fifth day a perfect "cast" of the larynx and trachea was expelled entire. This is—

(1.) Traumatic exudative laryngitis or traumatic croup.

(2.) Simple exudative laryngitis or *true croup* is a local inflammation in which an exudation develops varying in extent and consistence according to the intensity of the factor, which is principally atmospheric, *e. g.*, exposure to keen northeasterly winds, together with a constitutional state not accurately determined, which appears to predispose certain families and certain members of a family to this form of inflammation. This is the analogue in children to œdematous laryngitis in adults, and it bears the same relation to diphtheria as enteritis to enteric fever. The term "exudative" is preferred to "membranous" because the exudate in milder cases may be only mucus or pultaceous.

(3.) Secondary exudative laryngitis is an inflammation which occurs as a result of some of the exanthemata, as typhus and typhoid. "All of these three classes of membranous inflammation are mainly distinguished by the conspicous absence of certain grave constitutional symptoms ands equelæ, from—

(4.) Diphtheritic exudative laryngitis or true diphtheria.

Whilst laryngeal phthisis is conceded to be generally *secondary* to pulmonary tuberculosis, the possible occurence of *primary* tuberculous laryngitis is considered established and competent evidence in support of this position is cited at length.

Syphilitic laryngitis, benign and malignant neoplasms, and neuroses of the larynx all receive detailed consideration. Chapters XXIV and XXV are devoted to nasal and naso-pharyngeal affections, and Chapter XXVI to "Aural Maladies Associated with Naso-pharyngeal Disease." The limitation of space has necessitated the generalization of these morbid conditions under a classification of quite moderate extent. Nothing short of an additional volume could do justice to the subject.

The work is completed by a chapter of formulæ for remedies, and fifteen plates comprising 121 beautifully colored figures drawn from nature on the stone by Lennox Browne himself. To faithfully represent nature in color seems impossible, at least without an indefinite multiplication of stones in the lithographing process, but the drawing of these is accurate and the coloring is artistic and highly suggestive. It was the intention of the author to have them so arranged in binding that each plate could be opened out to lie beside the book for study during perusal of the text, thus avoiding the annoyance of constantly turning the leaves. But instead, the American publishers have bound the plates as ordinary leaves at the end of the book thus subjecting the readers to an inconvenience which detracts much from the profit and pleasure of reference. Otherwise the work of the publishers has been well done.

It is a book which will be read with gratitude to the author by every specialist on the subject, and one which meets the requirements of practitioners and students. W. E. CASSELBERRY.

THE PRINCIPLES AND PRACTICE OF MEDICINE. By the late CHARLES HILTON FAGGE, M. D., F. R. C. P. Including a section on cutaneous diseases, by P. H. Pye-Smith, M. D., and chapters on cardiac diseases, by Samuel Wilkes, M. D. Two vols. Pp. 1040, 883. Philadelphia: P. Blakiston Son & Co. Chicago: W. T. Keener.

This work embraces the whole subject of practical medicine including skin diseases.

The first impression on reading the book is of the scholarship of the author; to this is soon added that of his fairness. He appears to have no hobbies, and studies every subject for its facts wherever they may lead. This is shown specially in the account of the later advances in pathology given in the first 124 pages of the book. No better summary of the existing state of pathological knowledge in the same compass exists, certainly of the state of knowledge at the date at which it was written. But the book is something more than a judicious summary of the ideas of others. Keen and original observations appear on every page. The chapter on lithæmia, for instance, is full of suggestions to an American reader, who usually has so little experience with gout that its slighter manifestations are liable to escape his observation.

But in this country, at least, it would have been of advantage if he had included in the chapter a differential diagnosis between this state and that of chronic malarial toxæmia, which it resembles so closely in many respects.

The book seems to have been largely the outgrowth of the author's experience, and in parts where his experience has been limited it is incomplete. This same subject of malaria, for instance, is in point. The article on ague describes the ordinary and more obvious forms of the disease, but is sadly defective in the more subtle manifestations. Civic malaria and chronic malarial toxæmia form a large part of the practice of physicians, in American cities at least, and in their more obscure manifestations are sources of perplexity to the skillful perhaps oftener than to the unskillful. But here, where to us the most help is needed, the book is silent.

In the chapter on cholera, few will agree with the author in any of the bearings of the statement that "almost the only morbid state which can be mistaken for cholera is that produced by the administration of arsenic." On the contrary, the ordinary symptoms of acute arsenical poisoning are not those of cholera but of acute gastritis. Only in anomalous cases, mere medical curiosities, is there any resemblance to cholera.

The ordinary symptoms of tartar-emetic poisoning more nearly resemble cholera than do those o arsenical poisoning.

On the other hand, ordinary cholera morbus may resemble choiera so closely as to deceive the most experienced, as all the older physicians in this city can testify.

It is remarkable that this statement of the author should have escaped the attention of the editor, since the recent discussion over the comma bacillus has called special attention to this difficulty of diagnosis. Strümpel says, speaking of cholera morbus, "formerly the distiction from true Asiatic cholera was occasionally quite difficult, and only possible by a study of the important etiological facts and by demonstrating the connection of the single case with other cases of undoubted cholera." This statement is in perfect accord with American experience.

The article on laryngology is as good as could be expected in a work on general medicine, but the descriptions are not minute enough to enable one unacquainted with the use of the instruments to learn their use, and certainly are too elementary for the expert. And what is singular in an article of this kind, it is without illustration, which greatly impairs its usefulness.

The examples which have been given are a fair average of the whole book; it has great merit, with some deficiencies in minor points which perhaps the author would have supplied if he had lived to complete the work.

As to the style, one who was hypercritical might say it was too diffuse and sometimes monotonous; good for a lecture, where it is enlivened and emphasized by the voice and presence of the speaker, but for reading, somewhat in need of pruning.

It is entirely without illustrations. Their use would have saved both reader and writer much trouble.

The book is not an elementary one or of uniform excellence, but taken as a whole it is the best contribution which has appeared in English for some time. LESTER CURTIS.

THE REFRACTION OF THE EYE. A MANUAL FOR Students. By GUSTAVUS HARTRIDGE, F. R. C. S. With ninety-six illustrations. Third edition. Philadelphia: P. Blakiston Son & Co. Chicago: W. T. Keener.

This is a book of 240 pages, which, as its title indicates, treats of the refraction of the eye, with the kindred subjects, asthenopia and strabismus. Thirty-four pages are devoted to retinoscopy, which is well presented; and considerable space is devoted to strabismus and the forms of asthenopia. In general, the subject-matter discused is clearly stated, but considering that it is the third edition and that "the whole has been carefully revised, some chapters partly rewritten," such indefinite phrases as the following ought to have been improved: "When the eyes are completely at rest, the optic axes are either parallel or, more usually, slightly divergent. The angle thus formed between the visual and the optic axis is called the anglea, and varies according to the refraction of the eye," p. 39. Again, p. 41, "The nearer an object, the more we have to converge and so also with the accommodation."

The subject of retinoscopy is very fully presented. The type is clear, and the book is well illustrated.

OPHTHALMIC SURGERY. By ROBERT BRUDENELL CARTER, F. R. C. S., and WILLIAM ADAMS FROST, F. R. C. S. Illustrated. Philadelphia: Lea Bros. & Co. Chicago: A. C. McClurg & Co.

This is one of a series of clinical manuals for practitioners and students of medicine. In consists 554 pages of closely printed matter, and embraces the whole subject of ophthalmic practice, including the subject of lenses and the color-tests.

The print is not very clear, and the binding and general appearance of the book is scarcely up to the customary standing of books emanating from that well-known publishing house.

INTUBATION OF THE LARYNX. By F. E. WAXHAM, M. D. Pp. xii and 110. Chicago: Charles Truax. 1888.

This book contains a short account of the history of the operation, a clear description of the instruments as first used and as now modified, and their use. A chapter on the anatomy of the larynx; an enumeration of the author's cases, and Dr. O'Dwyer's 100 cases. It is well written, the illustrations are fair, many of them excellent, and it is well printed on heavy glazed paper.

A PRACTICAL TREATISE ON DISEASES OF THE SKIN, for the use of Students and Practioners of Medicine. Second edition, thoroughly revised and enlarged. Pp. xix and 676. By JAMES NEVINS HYDE, A. M., M. D. Philadelphia: Lea Brothers & Co. 1888. Chicago: A. C. McClurg & Co.

The profession, as well as the author, is to be congratulated upon the demand for a second edition of this excellent treatise. The opportunity which this has afforded for a thorough revision has been embraced, and this edition may be relied upon as accurately representing the present state of knowledge upon the subject of which it treats. To those who are familiar with the first edition, or who are acquainted with its author, more need not be said. But as there may be some who have neither privilege, to them it may be said that the book is, as its title claims, eminently a practical treatise. Its style is clear, its descriptions easily understood, and its arrangement systematic. It is concise and complete. If a student or practitioner can have only one book upon this subject, he can not do better than to have this one. E. W.

THE LAWS OF HEREDITY. By GEORGE WILLIAMSON, M. D. Pp. 383. 12mo. Chicago: H. W. Palmer, Lock Box 532. 1887.

The author seems to have been fully conscious of the importance of his subject, in its bearing upon the future of the human race. The views which he entertains are radical in the extreme, and are very clearly explained. He maintains that at the moment of its birth, a child possesses the germ, at least, of every trait, physical and mental, which it will or ever can possess, and that subsequent training can not materially modify them. He also thinks that a mother can control and modify these traits at will during her pregnancy. That is, that every child at birth is an exact reproduction of every pronounced and protracted mental condition of its mother during its intra-uterine life. It is upon this theory that he accounts for the very striking differences sometimes observed between children of the same parents. It is frankly admitted that this theory places a burden of truly awful and overwhelming responsibility upon the mothers of the race, but he says that there is no escape from it. He claims that the male parent exerts no influence whatever upon the child except through the imagination of the mother.

The discussion is carried very fully into a consideration of the personal and social questions which the theory involves.

It ought to have an index. But this can be corrected in a second edition, which it is to be hoped will be called for; since it is capable of a great deal of good. It can hardly fail to increase the well-being and happiness of any growing family in which it receives the attention which it merits.

E. W.

A MANUAL OF THE MINOR GYNÆCOLOGICAL OPERATIONS. By J. HALLIDAY CROOM, M. D. First American, from the second Edinburg, edition. By Lewis S. McMurtry, A. M., M. D. Pp. xii and 228. 12mo. Philadelphia: Records, McMullin & Co., Limited. 1888.

The American editor says that the book is intended to furnish the "student and practitioner a brief, simple, and practical account of the more common gynæcological operations."

It is all of this, and it is further proper to say that the additions to the text made in the American edition enhance the value of the book, which is, upon the whole, an excellent manual in its proper field.

### BOOKS RECEIVED.

Essentials of Chemistry, etc. By. R. A. Witthaus, A. M., M. D.

The Year Book of Treatment for 1887.

A Compend of Human Physiology. By A. P. Brubaker, A. M., M. D.

A Manual of Diseases of the Nervous System. By W. R. Gowers, M. D., F. R. C. P.

The Surgical Diseases of the Genito-Urinary Organs. By E. L. Keyes, A. M., M. D.

Essays on Hysteria, Brain-Tumor, and Some other Causes of Nervous Disease. By Mary Putnam Jocobi, M. D.

Morrow's Atlas of Venereal and Skin Diseases. Fasciculi, 1, 2, 3.

Accidents and Emergencies. By Charles W. Dulles, M. D.

Ophthalmic Surgery. By R. B. Carter, F. R. C. S., and W. A. Frost, F. R. C. S.

Lesions of the Vagina and Pelvic Floor. By B. E. Hadra, M. D.

A Treatise on Dislocations. By Lewis A. Stimson, B. A., M. D.

A Practical Treatise on Diseases of the Skin. By James Nevins Hyde, A. M., M. D.

The Laws of Heredity. By George Williamson, M. D.

Dermatitis Venenata, an Account of External tants upon the Skin. By James C. White, M. D.

Clinical Atlas of Venereal and Skin Diseases. By Robert W. Taylor, M. D.

A Manual of the Minor Gynæcological Operations. By J. Halliday Croom, M. D.

Intubation of the Larynx. By F. E. Waxham, M. D.

Saint Bartholomew's Hospital Reports.

The Language of Medicine. By F. R. Campbell, A. M., M. D.

### PAMPHLETS RECEIVED.

Chloroform the Best of Anæsthetics. By Julian J. Chisholm, M. D.

Pneumonia; its Mortality and Treatment. By Henry Hartshorn, M. D.

An Aseptic Atmosphere et al. By David Prince, M. D.

The Pulley Method of Advancing the Rectus. By A. E. Prince, M. D.

The Extraction of Cataract as Influenced by Mycological Development. By A. E. Prince, M. D.

The Legal Aspect of Suicide. By Hon. Wm. N Ashman.

Food Laws. By Henry Leffman, M. D.

The Neural and Psycho-Neural Factor in Gynecic Disease. By C. H. Hughes, M. D.

Infant Feeding, Especially with Reference to Subjects with Infantile Eczema. By L. Duncan Bulkley, A. M., M. D.

Clinical Notes on Pruritus. By L. Duncan Bulkley, A. M., M. D.

Fourteenth Annual Report of the Superintendent of the Cincinnati Sanitarium, 1887.

The Ischiatic Crutch. By A. B. Judson, M. D.

The Uses of Adhesive Plaster in Orthopedic Surgery. By A. B. Judson, M. D.

The Higher Medical Education. By R. O. Beard, M. D.

The Results of Laparotomy for Acute Intestinal Obstruction. By B. Farquar Curtis, M. D.

Abstract of Proceedings of the Michigan State Board of Health, Meeting of April 10, 1888.

Proceedings and Adresses at a Sanitary Convention held at Traverse City, Michigan, August 24 and 25, 1887.

Vesico-Vaginal Fistula. By Reuben A. Vance, M. D.

Heart and Blood Vessels in the Young. By A. Jacobi, M. D.

Stricture of the Urethra. By Henry J. Reynolds, M. D.

A New Method in the Treatment of the Vegetable Parasitic Diseases of the Skin. By Henry J. Reynolds, M. D.

